# Psychometric Analysis of a Microenvironment Secondhand Smoke Exposure Questionnaire

**DOI:** 10.3390/ijerph18073753

**Published:** 2021-04-03

**Authors:** Teresa DeAtley, Suzanne M. Colby, Melissa A. Clark, Alexander Sokolovsky, Rachel L. Denlinger-Apte, Patricia A. Cioe, Rachel Cassidy, Eric C. Donny, Jennifer W. Tidey

**Affiliations:** 1 Department of Behavioral and Social Sciences, Brown University School of Public Health, Providence, RI 02903, USA; suzanne_colby@brown.edu (S.M.C.); alexander_sokolovsky@brown.edu (A.S.); patricia_cioe@brown.edu (P.A.C.); rachel_cassidy@brown.edu (R.C.); jennifer_tidey@brown.edu (J.W.T.); 2 Department of Health Services, Policy and Practice, Brown University School of Public Health, Providence, RI 02903, USA; melissa_clark@brown.edu; 3 Department of Social Sciences and Health Policy, Wake Forest School of Medicine, Winston-Salem, NC 27101, USA; rdenling@wakehealth.edu; 4 Baptist Comprehensive Cancer Center and Department of Physiology and Pharmacology, Wake Forest School of Medicine, Winston-Salem, NC 27101, USA; edonny@wakehealth.edu

**Keywords:** cigarette smoke exposure, environmental tobacco smoke assessments, environmental tobacco smoke exposure, risk assessment, psychometric analysis

## Abstract

Background: We conducted a psychometric analysis of an adapted secondhand smoke (SHS) questionnaire by testing the three-component structure of the original scale that measures SHS exposure in home, work and social environments. Methods: The 15-item questionnaire was administered to 839 daily smokers participating in a multi-site randomized controlled trial. Following parallel analysis, we conducted a confirmatory factor analysis specifying a three-factor structure. Cronbach’s alphas and fit indices were calculated to assess internal consistency. Criterion validity was assessed by comparing the Social environments subscale to the Brief Wisconsin Inventory of Smoking Dependence Motives Social/Environmental Goads subscale. Predicative validity of the questionnaire was assessed using linear regressions and tobacco biomarkers of harm; NNAL, expired carbon monoxide and total cotinine. Results: Five items did not load onto any factor and were dropped, resulting in a 10-item questionnaire. The Cronbach’s alphas were (0.86), (0.77) and (0.67) for the Work, Social, and Home subscales, respectively. The WISDM subscale was moderately correlated with scores on the Social subscale (r = 0.57, *p* < 0.001). The questionnaire demonstrated predictive validity of smoke exposure above individual’s own reported use as measured by cigarettes smoked per day. Conclusions: Three constructs emerged; results indicate that a shortened 10-item scale could be used in future studies.

## 1. Introduction

Voluntary and involuntary exposure to tobacco smoke is a known health hazard and preventable cause of death [1,2]. Sources of exposure to involuntary smoke, otherwise known as secondhand smoke (SHS), include smoke coming directly from a burning tobacco product (sidestream smoke) and smoke exhaled by a smoker (mainstream smoke) [3,4,5]. SHS contains thousands of chemicals, including hundreds known to be toxic or carcinogenic [5]. There is risk associated with all levels of smoke exposure; exposures have been linked to serious negative health consequences among smokers and non-smokers, including lung cancer, myocardial infarction, and stroke [4,5]. The term SHS exposure has largely replaced environmental tobacco smoke (ETS) exposure in the literature as it indicates the involuntary nature of the exposure, whereas use of the term environmental has a more ambient connotation [5].

### Secondhand Smoke Assessments

Reviews of SHS assessments have primarily focused on questionnaire-based epidemiological studies [6]. Subjective assessments of secondhand smoke exposure include interviews or self-administered questionnaires, which rely on respondents’ self-report. Interviews tend to be more thorough than self-administered questionnaires but are also relatively resource intensive. There are also objective methods of assessing secondhand smoke exposure (e.g., personal monitors, biological samples), that are more resource-intensive and costly than subjective assessments [7].

Standardized SHS questionnaires have been used to document exposure to SHS in venues such as the home, workplace, leisure, and transportation spaces [6]. Previous psychometric research has supported the validity of these assessment by documenting moderate correlations between self-reported SHS exposure and airborne nicotine concentrations [8]. In addition, relative levels of tobacco smoke exposure among smokers and non-smokers from SHS questionnaires have been validated using serum cotinine concentrations [9] and hair nicotine levels [10].

The Environmental Tobacco Smoke Questionnaire (ETSQ) developed by Nondahl et al. (2005) [9] was designed to specifically measure the extent of self-reported involuntary smoke exposure in several microenvironments such as the home, workplace and during social interactions. The original version of the ETSQ was validated by examining associations between questionnaire responses and serum cotinine (nicotine metabolite) measurements among smokers and non-smokers. Specifically, cotinine level was used to validate self-reported non-smoking status, and, among non-smokers, cotinine level was found to be associated with self-reported home, workplace, social, and overall SHS exposure [9]. The ETSQ was expanded to a 15-item questionnaire when it was included in a large multi-site randomized trial conducted by Donny et al. [11], as described in greater detail in the section below. The Donny et al. version of the ETSQ questionnaire has been used in several recent randomized controlled trials (NCT01681875; NCT02139930; NCT02019459); however, to our knowledge, psychometric properties of the scale have not been examined since its adaptation [11,12,13].

We sought to explore the psychometric properties of this recently expanded SHS questionnaire that measures smoke exposure in these spaces. In addition to validating the adapted scale given its current and ongoing use in randomized controlled trials, the authors opted to change the scale name to more accurately reflect the language currently used in the scientific literature.

In the current secondary data analysis, which draws upon a large sample of over 800 smokers included in a multi-site trial, [14] we conducted a confirmatory factor analysis of the adapted SHS scale, specifying work, social and home factors a priori based on results of a principal component analysis (PCA) and parallel analysis. Three factors were selected to align with the previously validated ETSQ. We also tested three dimensions for validating subjective questionnaires: criterion, construct and predictive validity [7].

## 2. Materials and Methods

### 2.1. Sample

This secondary data analysis was conducted using de-identified data collected from smokers (N = 839) across 10 U.S. study sites. Study participants were non-treatment-seeking daily smokers who were willing to use research cigarettes varying in nicotine content for a 6-week period. The adapted questionnaire and other measures used for the current validation study were collected during a baseline period prior to randomization. Other details about the study have been previously described [14].

### 2.2. Original Scale Constructs

The original scale developed by Nondahl et al. [9] consisted of 12 items designed to measure SHS exposure in the home, at work and in social environments (Table 1, original version). Three questions in the original version pertain to the Home construct, three questions pertain to the Social construct, and two questions pertain to the Work construct. The remaining four questions assessed smoking status of the respondent and frequency and duration of smoke exposure in the past 24 h.

As noted above, the ETSQ was adapted for use in a large multi-site randomized controlled trial conducted by Donny et al. [11] (see Table 1, “Donny Adapted version”). During this adaptation, several questions were dropped, and other questions were reworded, resulting in a 15-item questionnaire that retained the focus on SHS exposure in the home, at work, and in social environments. For example, the original scale (Table 1) asks how many people in the household smoke inside, including cigars and pipes, whereas the adapted version (Table 1) of the questionnaire asks separate questions about cigarette versus other combusted product use. Another change is that the original scale assesses daily cigarette use by others, excluding the respondent, whereas the adapted version includes the respondent’s smoking habits. To collect additional information pertinent to the home and social factors, three additional questions were added to the adapted version of the questionnaire that assess rules about smoking, such as if an individual experiences smoke exposure in their car, and about the quantity of smokers in a participant’s network (family, friends and coworkers).

A third difference between the original version and the adapted version is that response options for indicating past-day SHS exposure differ. The original scale (Table 1) includes several questions about exposure over the last 24 h, in addition to questions that measure overall exposure. Instead the adapted version (Table 1) of the questionnaire only asks for participants to estimate SHS overall in their home, work and social settings.

### 2.3. Data Analysis Plan

All analyses were conducted using STATA [15]. As a first step, we used parallel analyses [16] to determine the number of factors to retain in the 15-item SHS scale. Next, a Confirmatory Factor Analysis with varimax rotation was conducted, specifying the determined number of factors to retain. Factors were evaluated based on Eigenvalues above one and the difference between each factor was examined for its conceptual interpretability and labeled accordingly. Items that were above a threshold of (0.5) were retained for each factor. No items cross loaded onto any of the three factors. Internal consistency was calculated for the total score and for each subscale. Intercorrelations among the three subscale scores were computed. Our sample size (N = 839) was well above the common convention requiring at least 10–15 respondents per scale item when evaluating an instrument [17,18]. Fit statistics were estimated for the overall confirmatory model, only using questions that were retained from our confirmatory analysis. We adjusted for strong residual correlations between scale items based on modification indices.

### 2.4. Criterion Validity Using the WISDM Scale

The Brief Wisconsin Inventory of Smoking Dependence Motives (WISDM) scale [19] is a psychometrically validated abbreviated version of the longer WISDM scale [20] that measures theoretically derived smoking motives. The Brief WISDM scale includes a social/environmental goads subscale (i.e., social stimuli or contexts that increase motivation to smoke), which is the mean of three items (“Most of the people I spend time with are smokers”; “A lot of my friends or family smoke”; and “Most of my friends and acquaintances smoke”) [19]. Each item is answered on a 7-point Likert scale ranging from 1 = Not true of me at all to 7 = Extremely true of me. In this sample, scores ranged from 1–7 for each item. This subscale had an alpha, 0.953 and the score of the subscale was calculated using the mean score of items of that subscale. The correlation between the Social SHS subscale and the WISDM social/environmental goads subscale was computed. The Brief WISDM does not include subscales pertaining to exposure to smokers at home or at work, nor did the Donny et al. (2015) [14] trial include other measures of smoke exposure at work or at home, so we were unable to assess criterion validity for those subscales in the final questionnaire.

### 2.5. Incremental Validity

We fit a series of linear regression models evaluating the predictive validity of our scale for biological measures of smoke exposure and harm beyond the effect of self-reported behavioral tobacco exposure as indexed by cigarettes per day (CPD). 4-(methylnitrosamino)-1-(3-pyridyl)-1-butanol (NNAL) was selected as a measure of smoking toxicity. NNAL is the metabolite of 4-(methylnitrosamino)-1-(3-pyridyl)-1-butanone (NNK), which has been identified as a specific carcinogen based on extant evidence [21]. Saliva cotinine and expired carbon monoxide (CO) were selected as biological measures of nicotine and tobacco exposure, respectively. Levels of cotinine, CO, and NNAL beyond those accounted for by direct smoke exposure (as measured by cigarettes per day) should reflect SHS exposure and be correlated with scores from each of our subscales.

## 3. Results

### 3.1. Construct Validation

Parallel analysis suggested a three-factor structure; the three factors accounted for 49.7% of the total variation in the questionnaire. Factor I had a high Eigenvalue at 2.91, and Factors II and III had Eigenvalues of 2.65 and 1.39, respectively. We retained a three-factor structure (Work, Social, Home) and specified three factors for the subsequent Confirmatory Factor Analysis.

### 3.2. Eliminated Items

Five items did not load onto any of the three factors. Items 1, 2, 4 and 12 measure SHS exposure in the home but did not load onto Factor III, the Home subscale. Item 9 assesses whether the respondent permits smoking in their car, which is relevant to social exposure, but did not load onto any factor. The range of the Cronbach alpha did not differ for the overall scale with the elimination of these five items (0.66 for the final version vs. 0.66 for the adapted version). Among the eliminated items, one item was removed from the Social subscale and 4 were removed from the Home subscale.

### 3.3. Confirmatory Factory Analysis

Based on the results from the confirmatory factor analysis (see Table 2), three subscales were formed: the Work subscale, consisting of items 5, 6, 7, 15 (Factor I), a Social subscale, consisting of items 10, 11, 13, and 14 (Factor II), and a Home subscale, consisting of items 3 and 8 (Factor III). The resulting 10-item questionnaire had a Cronbach’s alpha of (0.66) indicating that the scale had acceptable internal consistency. The Kaiser-Meyer-Olkin (KMO) measure of sampling adequacy indicates that this scale had middling sampling adequacy KMO of <0.70. The KMO for the Donny 15-item CFA was 0.72 and 0.71 for the 10-item CFA. The Cronbach’s alpha for the 4-item Work subscale in the final questionnaire was high (0.86), while the Cronbach alpha for the 4-item Social subscale was slightly lower but still good (0.77). The alpha for the 2-item Home subscale was the lowest but acceptable (0.68). Prior to the elimination of the five items, the Cronbach’s alphas were 0.72 and 0.50 for the Social and Home subscales, respectively.

The intercorrelation of mean scores for the Social and Work subscale was r = 0.07, (*p* < 0.05), the intercorrelation between the Social and Home subscale scores was r = 0.16 (*p* < 0.001), and the intercorrelation between the Home and Work subscales was r= −0.07 (*p*= 0.053). These low intercorrelations indicate that the scales are modestly related to each other and that this scale measures three distinct sources of SHS exposure.

### 3.4. Criterion Validity and Incremental Validity

Scores on the Brief WISDM Social/Environmental Goads subscale were correlated with scores on the adapted Social subscale (r = 0.57 *p* < 0.001), which supports the concurrent validity of this factor. Results from the regression analyses show that our final questionnaire has incremental predictive value after controlling for exposure to secondhand smoke and participants’ own behavioral self-report of their smoking level as measured by cigarettes smoked per day. After controlling for the variance attributable to this set of covariates, the Work scale accounted for additional variance in expired CO (*p* = 0.041) and NNAL (*p* = 0.000) and the Social subscale accounted for additional variance in total cotinine (*p* = 0.084) (Table 3).

### 3.5. Fit Indices

Fit indices for our newly validated SHS questionnaire fell within an acceptable range. The Root Mean Squared Error Approximation (RMSEA) was below the cut-off of 0.08 at (0.059) and the Comparative Fit Index (CFI) and Tucker-Lewis Index (TLI) were excellent at 0.968 and 0.952, respectively. These fit statistics indicate that our psychometrically validated 10-item questionnaire has acceptable fit. Based on the results from the modification indices, there were high correlations between items 13 and 14 as well as items 10 and 11. Additional adjustments were made to control for the covariance of these items.

## 4. Discussion

Psychometric analyses identified the underlying structure of the final SHS questionnaire. The resulting instrument, which we name the Secondhand Smoke Microenvironment Questionnaire, comprises three factors consistent with the constructs measured by the original version developed by Nondahl et al. (2005): Social, Work, and Home SHS exposure. Subscale reliability, as indexed by internal consistency, ranged from acceptable to excellent. The three subscales have high face validity and predictive validity, and the concurrent validity of the Social subscale was supported. Future work should expand upon this evaluation, for example by examining test–retest reliability, and other aspects of concurrent validity.

Based on our analysis, the Home construct of the final questionnaire only includes two questions whereas the original scale included three questions, including measurement of use of other combustible tobacco products in the home. Our study excluded people who used other combusted products more than 9 days per month, which may explain why this question did not load onto the Home subscale in this psychometric analysis.

This confirmatory factor analysis highlights the need for validation of other subscales, including criterion validity and content validity. However, it also suggests that the 10-item version, excluding items 1, 2, 4, 9 and 12, is appropriate for use (Appendix A). On the Secondhand Smoke Microenvironment Questionnaire, the Home subscale consists of items 1 and 5, the Social subscale consists of items 6-9 and the Work subscale consists of items 2, 3, 4 and 10.

One limitation of the existing scale is that it does not capture SHS from other products such as e-cigarettes or cannabis. Use of such products has become common especially among young adults over the past few years since the scale was developed [22]. Future versions of this scale should consider adding smoke exposure from these other nicotine and combustible products to adequately capture additional dimensions of SHS exposure in microenvironments.

## 5. Conclusions

This psychometric study supports the utility of the Secondhand Smoke Microenvironment Questionnaire for assessing SHS exposure in the work, home, and social spheres. The confirmatory factor analysis supports the three-factor structure, and the factors are reliable, have high face and predictive validity, and are consistent with prior work. The three-factor Secondhand Smoke Microenvironment Questionnaire allows researchers to administer subscales only when applicable to their population of interest. For example, the Work subscale could be omitted if researchers were working with a population of unemployed individuals. SHS Microenvironment subscales can also be used to determine the extent to which SHS exposure in different spheres of an individual’s natural environment moderates the effects of smoking cessation treatment or makes it more difficult for high exposure populations to quit. Further validation of the SHS Secondhand Smoke Microenvironment questionnaire subscales is an important area of future work.

## Figures and Tables

**Table 1:** Comparison of secondhand smoke (SHS) questionnaire items.

Item Number	Nondahl: Original Questionnaire	Donny: Adapted Questionnaire	Secondhand Smoke Microenvironment Questionnaire
1	Do you currently smoke	# people who smoke cigarettes in your home ^1^	Cigarettes smoked per day in home
2	Average exposure time (work)	# of people who smoke cigars, little cigars or pipes in your home	Smoking rules (work)
3	# People that smoke in the same areas as you (work)	CPD in home	Tobacco smoke exposure (work)
4	# of people living in home (excluding self)	Other combustible in home	# People that smoke (work) in the same area as you
5	# of people who smoke inside the home, (excluding self)	Smoking rules (work)	Rules about smoking (home)
6	CPD in home (excluding self)	Tobacco smoke exposure (work)	Tobacco smoke exposure (social)
7	How often exposed to tobacco Smoke (social)	# People that smoke in the same areas as you (work)	Average time overall tobacco smoke exposure
8	Average exposure time (social)	Rules about smoking (home)	# friends smoke
9	# people smoking (social)	Rules about smoking (car)	Top five friends smoke
10	Last 24 h exposure	Tobacco smoke exposure (social)	# coworkers smoke
11	Last 24 h exposure time	Average time overall tobacco smoke exposure	
12	Last 24 h # of people	# Family members smoke	
13		# friends smoke	
14		Top five friends smoke	
15		# coworkers smoke	

^1^ # stands for number.

**Table 2:** Factor Analysis of the SHS Microenvironment questionnaire.

	Item	Factor I (Work)	Factor II (Social)	Factor III (Home)
1	Number people who smoke cigarettes in your home	−0.0058	0.1760	0.2258
2	Number of people who smoke cigars, little cigars or pipes in your home	0.0470	0.1525	0.2333
3	CPD in home	−0.0394	0.1073	0.7625
4	Other combustible in home	−0.0392	0.1203	0.2481
5	Smoking rules (work)	0.7534	−0.0713	−0.0788
6	Tobacco smoke exposure (work)	0.7194	0.0341	−0.0042
7	Number of people that smoke (work) in the same area as you	0.8365	0.0194	−0.0024
8	Rules about smoking (home)	−0.0496	0.0498	0.7208
9	Rules about smoking (car)	0.1503	−0.1944	0.0324
10	Tobacco smoke exposure (social)	0.0155	0.6432	0.0959
11	Average time overall tobacco smoke exposure	0.0684	0.5803	0.1014
12	Number of family members who smoke	0.0405	0.2134	0.1685
13	Number of friends who smoke	0.0338	0.7297	0.0554
14	Top five friends smoke	0.0247	0.7314	0.0601
15	Number of coworkers who smoke	0.7430	0.1251	−0.0192

**Table 3:** Linear regressions predicting SHS questionnaire validity beyond self-reported exposure of CPD.

Biomarker of Exposure	Predictors	Beta Coefficient	95% CI	*p*-Value
NNAL	CPD	0.015	0.01–0.02	0.000 *
	SHS Home Subscale	−0.029	−0.07–0.01	0.187
	SHS Work Subscale	0.092	0.05–0.13	0.000 *
	SHS Social Subscale	0.005	−0.04–0.05	0.791
Expired CO	CPD	0.37	0.30–0.44	0.000 *
	SHS Home Subscale	−0.49	−1.3–0.30	0.227
	SHS Work Subscale	0.80	0.03–1.58	0.041 *
	SHS Social Subscale	0.28	−0.49–1.1	0.473
Total Cotinine	CPD	83.74	64.83–102.70	0.000 *
	SHS Home Subscale	34.98	−174.76–244.72	0.743
	SHS Work Subscale	−98.10	−302.97–106.78	0.348
	SHS Social Subscale	180.11	−24.33–384.56	0.084

* *p*-value below 0.05.

## Data Availability

This study is considered secondary data analysis. No new data were created and data sharing is not applicable to this article.
